# Biodegradable Vascular Patches for Arterial Reconstruction: In Vitro Study and Long-Term Implantation in a Sheep Model

**DOI:** 10.3390/biomedicines14102383

**Published:** 2026-10-19

**Authors:** Ekaterina Prokudina, Eugenia Senokosova, Larisa Antonova, Eugenia Krivkina, Tatyana Akentyeva, Tatyana Glushkova, Vera Matveeva, Rinat Mukhamadiyarov, Andrey Mironov, Egor Sardin, Alexey Kolesnikov, Anastasia Arnt, Nikita Kochergin

**Affiliations:** 1 Department of Cardiovascular Surgery, Research Institute for Complex Issues of Cardiovascular Diseases, 650002 Kemerovo, Russia; antolv@kemcardio.ru (L.A.); miroav@kemcardio.ru (A.M.); sardes@kemcardio.ru (E.S.); koleau@kemcardio.ru (A.K.); arntaa@kemcardio.ru (A.A.); nikotwin@mail.ru (N.K.); 2 Department of Experimental Medicine, Research Institute for Complex Issues of Cardiovascular Diseases, 650002 Kemerovo, Russia; sergea@kemcardio.ru (E.S.); kriveo@kemcardio.ru (E.K.); akentn@kemcardio.ru (T.A.); glushtv@kemcardio.ru (T.G.); matvvg@kemcardio.ru (V.M.); muhara@kemcardio.ru (R.M.)

**Keywords:** vascular patch, biodegradation, silk fibroin, bovine pericardium, electrospinning, biocompatibility, sheep

## Abstract

**Background/Objectives**: The development of biodegradable materials for cardiovascular surgery that promote gradual replacement with native tissue is a key scientific challenge. Silk fibroin (SF) is a naturally derived biodegradable material with low immunogenicity and good biocompatibility. This study compared the in vitro and in vivo properties of SF patches with bovine pericardium (BP). **Methods**: SF and BP patches were characterized for ultrastructure, physico-mechanical properties, hydrolytic degradation, protein adsorption, hemocompatibility, and in vitro biocompatibility. SF and BP patches were implanted into sheep carotid arteries for 2, 6, and 12 months, with intravital ultrasound and optical coherence tomography (OCT) monitoring, followed by morphological examination. **Results**: The SF patch exhibited a porous-fibrous structure, uniaxial tensile properties similar to those of native arteries, and resistance to in vitro hydrolytic degradation for 12 months. It caused no hemolysis, minimal platelet aggregation, and in vitro biocompatibility comparable to BP. At 2 months of implantation, both types of patches showed no stenosis, aneurysms, or calcification, with endothelial-like cell coverage. At 6 months, SF patches displayed minimal degradation, no calcification, and no neointimal hyperplasia (NH), whereas BP patches showed delamination and NH. At 12 months, NH was observed in both groups; the degrading SF patch was partially replaced by host tissue, maintaining a three-component tissue organization comprising neointima, residual patch material, and neoadventitia, whereas the BP patches exhibited focal calcification. **Conclusions**: The SF patch showed promising in vitro and in vivo performance, artery-like tensile properties, and partial hemocompatibility. However, elastin absence and 12-month NH warrant further optimization of the material.

## 1. Introduction

The use of carotid endarterectomy for correcting significant carotid artery stenosis substantially reduces the risk of stroke; however, the choice of material for closing the arteriotomy defect critically affects long-term vessel patency [1]. The use of autologous vein patches is often limited, whereas synthetic patches (ePTFE, Dacron) provoke neointimal hyperplasia (NH) at the anastomosis [2]. A clinically attractive alternative is xenopericardium; however, it is non-resorbable and susceptible to late calcification [3]. Consequently, the development of patches from biodegradable materials that undergo remodeling into tissue resembling native vasculature remains an important goal for regenerative medicine and vascular surgery.

For vascular patches, biostable materials (e.g., xenopericardium) resist degradation but do not support active remodeling. In contrast, biodegradable polymers—such as polylactide or polycaprolactone—can be replaced by host tissue, yet many lack sufficient mechanical strength under arterial pressure [4]. The challenge is to combine adequate initial strength with controlled degradation and favorable host response.

None of the currently available biomaterials fully satisfy the demands for a carotid patch, especially regarding long-term blood contact. Premature degradation triggered by the host inflammatory response can lead to structural failure [5]. Concurrently, calcification—calcium phosphate deposition within the material—compromises mechanical integrity and biocompatibility [6]. Therefore, a comprehensive evaluation of physico-mechanical properties, hemocompatibility, and in vivo remodeling is mandatory.

We selected silk fibroin (SF) from *Bombyx mori* as a biodegradable, natural-origin material. After removing the sericin coat (“degumming”), purified SF can be processed into various architectures (e.g., fibrous matrices) that support cell adhesion and proliferation [7,8]. Unlike many synthetic polymers, SF exhibits high tensile strength and slow degradation, making it a candidate for arterial patch applications [9].

As a biostable reference, we used bovine pericardium (BP)—a clinically established patch material for carotid artery reconstruction. BP has good properties and initial hemocompatibility, but it does not undergo remodeling. Therefore, we hypothesized that the SF patch would match BP in short-term performance and in long-term remodeling and the absence of calcification.

Despite growing interest in electrospun SF for vascular applications, several knowledge gaps remain. Most studies are limited to short-term in vitro or brief in vivo follow-up (1–6 months), limiting insight into long-term degradation and remodeling [10]. Comparisons with clinical standards like BP are scarce, and longitudinal imaging of SF grafts is lacking. This study provides: (1) a 12-month in vivo evaluation of SF patches in sheep carotids; (2) ultrasound and OCT monitoring; (3) direct comparison with BP; and (4) integrated in vitro–in vivo analysis.

In this study, we therefore tested the hypothesis that the SF patch would demonstrate mechanical, hemocompatible, and cytocompatible properties comparable to those of the BP patch, while providing in vivo remodeling and functional patency. To test this, we: (1) characterized the physicochemical and uniaxial tensile properties of both patches; (2) assessed platelet adhesion and hemolysis in vitro; (3) assessed cytocompatibility using endothelial cell line Ea.hy 926; (4) implanted patches into sheep carotid arteries for 2, 6, and 12 months; (5) conducted in vivo studies including ultrasound to monitor vascular patency and blood flow parameters and intravascular optical coherence tomography (OCT) to assess patch remodeling, neointimal coverage, and functional integrity; and (6) performed histomorphology analysis of explanted patches.

## 2. Materials and Methods

### 2.1. Isolation and Purification of SF from Cocoons of Bombyx mori

The purification process began with the degumming of *Bombyx mori* cocoon fragments (ø < 0.5 cm). This involved two 30 min cycles of boiling in a 0.02 M Na_2_CO_3_ (Mikhailovsky Factory of Chemical Reagents, Barnaul, Russia), each followed by extensive rinsing with dH_2_O (Figure 1). Subsequently, the dried SF product was dissolved in 9.3 M LiBr (CAS No. 7550-35-8, Central Drug House Ltd., Darya Ganj, New Delhi, India) at 60 °C over 4 h. The resulting solution was then purified via dialysis against dH_2_O for two days using a 3.5 kDa MWCO membrane (BS-QT-022, Biosharp, Anhui, China).

The aqueous SF solution was centrifuged twice at 12,700 *g* at 4 °C for 20 min using a Microfuge 20R ultracentrifuge (Beckman Coulter, Inc., Brea, CA, USA). The resulting SF solution was poured into molds, frozen at −60 °C, and lyophilized (FreeZone Benchtop Freeze Dryer, Labconco Corporation, Kansas City, MO, USA). The SF solution concentration was calculated using the following formula in 8 replicates:

(1) C = 100 × M/V,

where C—the SF concentration (%, wt/volume); M—the dry weight of SF (g); and V—the volume of SF before lyophilization (ml).

### 2.2. Preparation of SF-Based Biodegradable Patches and BP Control

To obtain biodegradable patches, we first dissolved SF in HFIP at a concentration of 15% (*w*/*v*) (Figure 1). The solution was then electrospun using a Nanon-01A (MECC CO., LTD., Ogori-shi, Fukuoka, Japan) system under the following conditions: 20 kV voltage, 1 mL/h feed rate, 15 cm needle-to-collector distance, and a rotating mandrel (8 mm diameter, 200 rpm) (Figure 1). A 22G needle served as the spinneret. Immediately after collection, the scaffolds were stabilized by soaking in 98% ethanol for 20 min, which induced the conversion of SF from an amorphous to a β-sheet-rich water-insoluble form [11]. As a non-degradable comparator, we used clinically approved epoxy-treated BP patches (NeoKor, Kemerovo, Russia).

### 2.3. Scanning Electron Microscopy of Surface Morphology and Fiber Architecture

The surface morphology and fibrous structure of the patches were examined using scanning electron microscopy (SEM). Specimens measuring 0.25 cm^2^ (n = 6 specimens per material) were cut from each patch and coated with a 15 nm thick silver-palladium (Ag-Pd) layer using an ACE200 EM sputter coater (Leica Mikrosysteme GmbH, Vienna, Austria). Imaging was performed with an S-3400N scanning electron microscope (Hitachi, Tokyo, Japan) operated in high-vacuum mode at an accelerating voltage of 15 kV.

### 2.4. Uniaxial Tensile Testing

Mechanical parameters of the patches were assessed under uniaxial tension using a Zwick/Roell Z-series universal testing machine (ZwickRoell GmbH & Co. KG, Ulm, Germany) equipped with a 50 N load cell and operated at a crosshead speed of 50 mm/min. Rectangular specimens (n = 6 specimens per material) were cut longitudinally from each patch. Prior to testing, all samples were pre-wetted by immersion in physiological saline (0.9% NaCl) for 30 min at room temperature to simulate the hydrated state in vivo.

The following parameters were recorded: maximum tensile stress (MPa), force at failure (Fmax, N), relative elongation at break (%, as a measure of material elasticity), and Young’s modulus (MPa, reflecting material stiffness).

BP-patches served as the non-degradable comparator. Additionally, native vascular tissue references were tested, including sheep carotid artery (*a. carotis*) and human internal mammary artery (*a. mammaria*); *a. mammaria* was used as a clinical reference (the gold standard for coronary bypass grafting) and *a. carotis* was used as an anatomical reference. Human tissue was obtained from patients undergoing coronary artery bypass grafting after written informed consent.

### 2.5. In Vitro Hydrolytic Degradation

Degradation behavior of the SF-patches was evaluated under simulated physiological conditions. Specimens measuring 0.25 cm^2^ were cut from each patch and weighed using a GR-200 analytical balance (A&D Company, Limited, Tokyo, Japan). Under sterile conditions, each sample was placed in a sealed sterile vial containing 2.5 mL of sterile saline solution (0.9% NaCl, pH 7.4) and incubated at 37 °C for predetermined time points: 1, 3, 6, and 12 months (n = 6 specimens per time point).

At the end of each incubation period, the specimens were retrieved from the vials, dried completely, and re-weighed. The pH of the residual incubation solution was measured using a HANNA Instruments pH211R meter (Hanna Instruments Deutschland GmbH, Vöhringen, Germany).

Degradation was quantified by calculating the percentage mass loss relative to the initial weight. Concurrently, any shift in pH of the surrounding medium was recorded as an indirect indicator of acidic degradation byproducts.

### 2.6. Protein Adsorption Assay

The affinity of the patch materials for blood plasma proteins was evaluated using albumin and fibrinogen as model proteins. Specimens of both SF and BP patches (1 cm^2^ each, n = 6 specimens per material) were first rinsed with ethanol and air-dried. Separate stock solutions of bovine serum albumin (BSA) and fibrinogen were prepared at a concentration of 1 mg/mL in phosphate-buffered saline (PBS). Each dried patch sample was then incubated in 1 mL of either protein solution for 2 h at 37 °C.

After incubation, the samples were washed successively with PBS and deionized water to remove non-adherent proteins. Subsequently, the adsorbed proteins were eluted by immersing each sample in 1 mL of 2% sodium dodecyl sulfate (SDS) solution for 2 h at 37 °C. The protein concentration in the SDS eluate was quantified using a Micro BCA™ protein assay kit (ab207003, Abcam, Cambridge, UK) with a Genesys 6 spectrophotometer (Thermo Scientific, Waltham, MA, USA) at 562 nm. The total amount of adsorbed protein (expressed in mg) was calculated per 1 mL of eluate.

### 2.7. Hemocompatibility Evaluation

The blood compatibility of SF and BP patches was assessed by measuring hemolysis, platelet aggregation and adhesion following contact with the material.

#### 2.7.1. Hemolysis Assay

Hemolysis induced by the patch materials was quantified using fresh citrated blood. Specimens of each patch type (25 cm^2^, n = 6 specimens per material) were placed in cuvettes containing 10 mL of saline (0.9% NaCl) and pre-incubated for 2 h at 37 °C. Subsequently, 200 μL of fresh citrated blood was added to each cuvette, mixed gently, and incubated for another 60 min at 37 °C. Saline and distilled water served as negative (0% hemolysis) and positive (100% hemolysis) controls, respectively. After incubation, the liquid from each cuvette was transferred to test tubes and centrifuged at 2800 rpm for 10 min to sediment intact red blood cells. The optical density (OD) of the supernatant was measured at 545 nm using a GENESYS 6 spectrophotometer (Thermo Scientific, USA). The percentage of hemolysis was calculated according to the formula

(2) H (%) = (OD_test_ − OD_neg_)/(OD_pos_ − OD_neg_) × 100,

where OD_test_ is the optical density of the sample incubated with the patch, OD_neg_ is the OD of the negative control (saline, no hemolysis), and OD_pos_ is the OD of the positive control (distilled water, complete hemolysis) [12].

#### 2.7.2. Platelet Aggregation

Whole blood from healthy donors was collected into 3.8% sodium citrate (9:1 blood:citrate ratio) and centrifuged at 1000 rpm for 10 min to obtain platelet-rich plasma (PRP). Platelet-poor plasma (PPP) was prepared by further centrifuging an aliquot of PRP at 4000 rpm for 20 min and used for baseline calibration. Patch specimens (0.25 cm^2^, n = 6 specimens per material) were placed in cuvettes containing PRP for 3 min. Aggregation was then triggered by adding adenosine diphosphate (ADP, AGRENAM AG-6) at a final concentration of 20 μM. The maximum aggregation percentage was recorded after 5 min using a semi-automated four-channel aggregometer (ARAST 4004, LABiTec, Ahrensburg, Germany).

#### 2.7.3. Platelet Adhesion

To evaluate platelet adhesion on the patch surfaces, specimens (0.25 cm^2^, n = 6 specimens per group) were incubated with 300 μL of PRP for 1 h at 37 °C. After incubation, non-adherent platelets were removed by washing with PBS (pH 7.4), and the samples were fixed in 4% paraformaldehyde for 10 min. The fixed specimens were then incubated overnight (12 h) at 4 °C with primary antibodies: rabbit anti-CD41 (ab134131, Abcam, UK) and mouse anti-CD62P (ab54427, Abcam, UK). After washing with PBS containing 0.1% Tween 20, the samples were incubated for 1 h at room temperature with secondary antibodies: Alexa Fluor 488-conjugated goat anti-rabbit IgG (A11034, Thermo Fisher Scientific, Waltham, MA, USA) and Alexa Fluor 555-conjugated goat anti-mouse IgG (A31570, Thermo Fisher Scientific, USA). Following another wash with PBS/Tween 20, the stained samples were examined using a confocal laser scanning microscope LSM700 (Carl Zeiss, Jena, Germany).

### 2.8. In Vitro Biocompatibility Characterization

The biocompatibility of the SF and BP patches was evaluated in vitro through two complementary approaches: (i) assessment of cytocompatibility using a vascular endothelial cell line, and (ii) characterization of cell–material adhesive interactions via quantification of focal adhesion proteins and cytoskeletal organization.

#### 2.8.1. Endothelial Cell Culture and Seeding

The Ea.hy 926 endothelial cell line was maintained in DMEM/F12 medium (11320033, Thermo Fisher Scientific, USA) supplemented with HAT (H0262, Sigma-Aldrich, St. Louis, MO, USA), 10% fetal bovine serum (26140079, Thermo Fisher Scientific, USA), antibiotics (0378016, Thermo Fisher Scientific, USA), and amphotericin B (15290018, Thermo Fisher Scientific, USA). Cells were passaged upon reaching 70% confluence and detached using 0.025% trypsin-EDTA solution (15400054, Thermo Fisher Scientific, USA). All cultures were maintained under sterile conditions in a humidified incubator at 37 °C with 5% CO_2_.

For each experiment (Viability Assay and Assessment of Adhesive Properties), circular patch specimens (ø 1.6 cm, n = 2 specimens per group) were secured to the bottom of 24-well culture plates. ECs were seeded onto the patches at a density of 30,000 cells per well and cultured in complete growth medium for 72 h. Control wells consisted of tissue culture plastic (TCP) seeded with the same number of cells and incubated under identical conditions, without any patch material.

#### 2.8.2. Viability Assay

After 72 h of culture, cell viability on each substrate was assessed using a dual-fluorescence staining protocol. The cells were incubated with Hoechst 33342 (10 µg/mL, 14533, Sigma-Aldrich, USA) for 10 min to label all nuclei, followed by ethidium bromide (30 µg/mL, 46067, Sigma-Aldrich, USA) for 1 min to selectively stain non-viable cells. Fluorescence imaging was performed using an Axio Observer Z1 inverted microscope (Carl Zeiss, Germany) with excitation/emission wavelengths of 350/461 nm for Hoechst and 546 nm for ethidium bromide. For each replicate, ten random fields of view were captured, and cell counts were normalized to 1 mm^2^ of patch surface area. The percentage of dead cells was calculated as

(3) Dead cells (%) = N_dead_/N_total_ × 100,

where N_dead_ is the number of ethidium-positive (red) nuclei, and N_total_ is the total number of Hoechst-positive (blue) nuclei. The proportion of viable cells was then determined by subtracting the percentage of dead cells from 100%.

#### 2.8.3. Assessment of Adhesive Properties (Talin and F-Actin)

To evaluate the adhesive phenotype of EC on the patch surfaces, the area occupied by Talin-positive focal adhesions and the organization of the actin cytoskeleton were analyzed. After 72 h of culture, the specimens were fixed in 4% paraformaldehyde for 10 min and permeabilized with 0.1% Triton X-100 for 15 min. Non-specific binding was blocked by incubation with 1% BSA in PBS for 1 h at room temperature.

The samples were then incubated overnight at 4 °C with primary antibodies against Talin (rabbit polyclonal, ab71333, Abcam, UK). Following washing with PBS, the specimens were incubated for 1.5 h at room temperature with secondary antibodies (Donkey anti-Rabbit IgG (H+L) Alexa Fluor 488 conjugate, A21206, Thermo Fisher Scientific, USA; diluted 1:600) and simultaneously with Phalloidin Alexa Fluor 568 (A12380, Invitrogen, Carlsbad, CA, USA) to visualize filamentous actin (F-actin). Nuclei were counterstained with DAPI (10 µg/mL, D9542, Sigma-Aldrich, USA) for 40 min. After final washes, the stained samples were mounted on glass slides using ProLong mounting medium (P36930, Life Technologies, Carlsbad, CA, USA) and examined using a confocal laser scanning microscope LSM 700 (Carl Zeiss, Jena, Germany).

### 2.9. Surgical Implantation and In Vivo Evaluation

The in vivo performance of the SF and BP vascular patches was evaluated using a sheep carotid artery model. A total of 18 healthy, non-pregnant female sheep weighing 42–45 kg were used in this study. The animals were divided into three observation periods (2, 6, and 12 months), with 6 animals per period. Each animal received an SF patch in one carotid artery and a BP patch in the contralateral carotid artery. Thus, for each time point, the SF and BP groups each comprised n = 6 patches (one per animal). The total number of implanted patches for both groups at all time points was 36. All surgical and analytical procedures were performed by personnel blinded to the group allocation.

The animals were housed under standard farm conditions with ad libitum access to food and water, and received routine veterinary care throughout the experimental period. All surgical procedures and animal handling were conducted in compliance with the European Convention for the Protection of Vertebrate Animals used for Experimental and other Scientific Purposes and the Declaration of Helsinki. The experimental protocol received formal approval from the Local Ethics Committee of the Federal State Budgetary Scientific Institution “Research Institute for Complex Issues of Cardiovascular Diseases” (Protocol No. 1, dated 26 January 2024).

Rectangular patch specimens (SF and BP) measuring 40.0 mm × 4.0 mm were implanted into the carotid arteries of the animals. The total follow-up period was set at 12 months, with scheduled intermediate endpoints at 2, 6, and 12 months post-implantation for intravital imaging assessments.

Postoperative surveillance of vessel patency was performed using a portable color Doppler ultrasound system (M7, Mindray, Shenzhen, China). Ultrasound examinations were performed at 1 and 5 days after surgery, and then at the terminal time points corresponding to each animal group (e.g., 2, 6, and 12 months).

For high-resolution intravital imaging of patch remodeling, OCT was performed via a transcarotid approach at the predetermined endpoints (2, 6, and 12 months) using an ILUMIEN system (Abbott Vascular, Westford, MA, USA) with the DragonflyTM OPTISTM Imaging Catheter (Abbott Vascular, USA). A 5 cm segment of the carotid artery encompassing the implanted patch was scanned with the OCT transducer at a pullback speed of 25 mm/s. To ensure optimal image quality and clear visualization of the vessel lumen, 25 mL of radiographic contrast agent was infused at a rate of 10 mL/s immediately prior to image acquisition.

### 2.10. Histological Processing of Explanted Specimens

At the predetermined endpoints (2, 6, and 12 months after implantation), the animals were euthanized, and the carotid artery segments bearing the SF or BP patches, together with adjacent proximal and distal tissue margins, were surgically retrieved. The harvested explants were fixed, dehydrated, and embedded in paraffin blocks following standard histological procedures. Serial sections (4–5 µm thick) were cut and subjected to three staining protocols: hematoxylin and eosin (H&E) for general tissue architecture, van Gieson’s stain for collagen fiber assessment, and alizarin red C for detection of calcium deposits. The detailed staining protocols have been described in our earlier publication [13].

The stained sections were examined under an AXIO Imager A1 light microscope (Carl Zeiss, Germany) at objective magnifications of ×50, ×100, and ×200.

### 2.11. Immunofluorescence Staining and Confocal Microscopy

The cellular and extracellular matrix composition of the explanted SF and BP patches was characterized by immunofluorescence staining following a previously established protocol [13]. Briefly, cryosections were rehydrated and subjected to antigen retrieval if required. To block nonspecific binding, sections were incubated with appropriate blocking serum. The following primary antibodies were applied overnight at 4 °C: rabbit anti-CD31 (endothelial cell marker, ab28364, Abcam, UK), mouse anti-α-smooth muscle actin (α-SMA, smooth muscle cell marker, ab7817, Abcam, UK), rabbit anti-von Willebrand factor (vWF, endothelial marker, ab6994, Abcam, UK), rabbit anti-collagen type IV (basement membrane component, ab6586, Abcam, UK), mouse anti-collagen type I (interstitial matrix marker, ab23446, Abcam, UK), rabbit anti-collagen type III (reticular fibers, NB600-594, Novus Biologicals, Centennial, CO, USA), and mouse anti-elastin (elastic fiber component, E4013, Sigma-Aldrich, USA).

After washing with PBS, the sections were incubated for 1.5 h at room temperature with secondary antibodies: Alexa Fluor 488-conjugated goat anti-rabbit IgG (A11034, Thermo Fisher Scientific, USA) and Alexa Fluor 555-conjugated goat anti-mouse IgG (A31570, Thermo Fisher Scientific, USA). To eliminate tissue autofluorescence, the sections were treated with Autofluorescence Eliminator Reagent (EMD Millipore Corporation, Temecula, CA, USA) prior to staining. Nuclei were counterstained with DAPI (10 µg/mL, Sigma-Aldrich, USA) for 5 min. Finally, the stained slides were mounted with coverslips using ProLong mounting medium (Thermo Fisher Scientific, USA).

Fluorescence images were acquired using an LSM 700 laser scanning confocal microscope (Carl Zeiss, Germany) with appropriate excitation/emission filters for each fluorophore. The distribution and intensity of each marker were analyzed qualitatively to assess endothelialization, smooth muscle cell infiltration, and extracellular matrix remodeling within the patches.

### 2.12. Statistics

Data were analyzed using GraphPad Prism version 8.0 (GraphPad Software, San Diego, CA, USA). The sample size for the in vivo experiments was determined based on the principles of the 3Rs and the expected effect size observed in a pilot study, with n = 6 animals per group per time point (a total of 18 animals: 6 animals × 3 time points). For in vitro biocompatibility assays, n = 2 samples per group were used, which is consistent with exploratory cytocompatibility testing. No formal power analysis was performed due to the exploratory nature of the study.

For all parameters determined by microscopy methods (immunofluorescence, confocal microscopy, scanning electron microscopy), measurement results obtained from 10 non-overlapping fields of view for each sample were averaged prior to statistical analysis, such that a single value corresponded to each biological sample.

The Kolmogorov–Smirnov test checked normality. Between two groups, the Mann–Whitney U test was used; for three or more groups, Kruskal–Wallis with Dunn’s post hoc test was applied. Significance was set at *p* < 0.05. Results are medians [Q1; Q3].

## 3. Results

The isolation and purification of SF were carried out according to an adapted protocol presented by L. Yu-Ting et al. [11]. A brief scheme for the isolation of SF is shown in Figure 1. The final product of this type of isolation was lyophilized SF sponges. The concentration of the aqueous SF solution obtained was 6.07 ± 0.56%, which is consistent with the data of L. Yu-Ting et al. [11].

Electrospinning from a 15% SF solution in HFIP under the above parameters produced SF patches with an acceptable porous-fibrous structure.

### 3.1. Surface Morphology of the Patch Materials

The ultrastructural features of the fabricated vascular patches were examined by SEM. The SF scaffold exhibited a fibrous surface morphology characterized by densely compacted, flattened fibres with multiple fusion points at fibre intersections and only occasional surface pores (Figure 2a). The mean fibre diameter was determined to be 4.8 ± 1.4 µm.

By contrast, the BP patch preserved its native extracellular matrix organization, displaying a distinctive textured surface arising from the wavy orientation of collagen bundles (Figure 2b). The sparse porosity observed on the BP surface is attributable to the tight packing of its constituent collagen fibrils.

Overall, the SF patches possessed a more open and porous framework compared to the BP counterparts. This structural advantage is expected to facilitate efficient EC seeding and support subsequent patch remodelling following implantation into the vascular wall.

### 3.2. Tensile Mechanical Properties

Following ethanol treatment and subsequent drying, the SF patches became stiff and lost their inherent flexibility. However, immersion in saline or water fully restored their original compliance. Consequently, all subsequent mechanical tests were performed on hydrated specimens.

In terms of maximum tensile stress and force at failure (Fmax), the SF patches exhibited values comparable to those of native arterial references (mammary and carotid arteries), whereas the BP material displayed significantly higher values for both parameters (Figure 3a,b). The elastic properties of the SF, expressed as relative elongation at break, fell between those of the two native vessels: it was 2.8-fold lower than that of the carotid artery, but 2.3-fold higher than that of the mammary artery (Figure 3c). Its Young’s modulus, accordingly, lay within the range of values recorded for native arterial tissues (Figure 3d).

The BP patches, in contrast, showed mechanical behaviour markedly different from that of native vessels (Figure 3a–d). They exhibited the highest ultimate tensile strength and tear resistance of all groups tested (Figure 3a,b). However, their elasticity was nearly twofold lower than that of the carotid artery, yet 3.5-fold greater than that of the mammary artery (Figure 3c).

Taken together, among the tested materials, the SF patches most closely resembled native arterial tissue in their uniaxial tensile properties. However, as only uniaxial tensile testing was performed, these findings should be considered as an initial mechanical screening rather than a comprehensive assessment of suitability for arterial reconstruction.

### 3.3. Hydrolytic Stability

The hydrolytic stability of the ethanol-cross-linked SF patches was evaluated, given their biodegradable polymer composition and porous fibrous architecture, which could potentially compromise this property [8]. BP patches were not included in this analysis, as their inherent stability in aqueous environments rendered such assessment unnecessary.

The SF patches exhibited resistance to hydrolytic degradation, with only a minor reduction in dry mass observed over the 12-month incubation period in saline solution. The maximum mass loss recorded was 5.4 [3.1; 7.3] % of the initial weight, which occurred at the three-month time point. Notably, no further substantial degradation was detected beyond this period: the additional mass loss between 3 and 12 months remained below 1% of the original mass.

Although the SF patches demonstrated overall hydrolytic stability, their degradation was accompanied by a gradual acidification of the incubation medium. The pH decreased from the initial value of 7.4 to a minimum of 6.56 after 12 months of incubation, indicating the release of acidic degradation by-products.

### 3.4. Protein Adsorption on Patch Surfaces

Quantitative analysis of protein adsorption revealed that the SF patches exhibited substantially higher binding capacity for both model proteins compared to the BP material (Table 1). Specifically, the amount of albumin eluted from SF was approximately twofold greater than that from BP, while fibrinogen adsorption was nearly sixfold higher on SF than on BP.

### 3.5. Hemocompatibility Assessment

The hemolysis assay revealed that the SF patches induced significantly lower hemolysis compared to the BP patches, with a 3.3-fold reduction in the degree of hemolysis (Table 2). Both materials, however, remained within the acceptable limits for hemocompatibility, as their hemolysis percentages did not exceed the maximum permissible values reported in the literature [14].

Platelet aggregation, triggered by ADP after contact with the patch materials, was comparable between SF and BP and did not exceed the level observed in PRP controls (Table 2), suggesting that neither material promotes excessive platelet activation.

In contrast, the adhesion and activation of platelets on the SF patches were significantly lower than on the BP surfaces, as assessed by confocal microscopy of CD41-positive and CD62p-positive adherent cells (Figure 4a,b).

### 3.6. In Vitro Biocompatibility of Patch Materials

The biocompatibility of the SF and BP patches was evaluated in vitro using endothelial cell line Ea.hy 926 cultured on the materials for 72 h. Initial cell attachment was comparable between the two patch types, with similar cell densities after 3 days (median: 105.7 cells/mm^2^ for both). However, this value represented only approximately 25% of the density achieved on TCP (444.2 cells/mm^2^) (Figure 5a).

Cell viability, assessed by dual fluorescence staining (Hoechst 33342/ethidium bromide), was high on SF and did not differ significantly from the plastic control. In contrast, viability on BP was lower than on the control (Figure 5b).

To characterize cell–surface interactions, we examined the adhesive morphology of EC via immunofluorescence staining for Talin (focal adhesion marker) and F-actin (cytoskeletal organization). This analysis revealed material-dependent differences in cell spreading and adhesion complex formation (Figure 5c,d). On the plastic control, cells formed a confluent monolayer with typical cobblestone morphology and tight intercellular junctions. The largest Talin-positive focal adhesion areas were observed on plastic, where they correlated with dense F-actin bundles (Figure 5c). In contrast, neither SF nor BP supported complete monolayer formation.

On SF patches, cells exhibited larger and more discrete Talin/F-actin clusters than on BP, suggesting more robust integrin-mediated adhesion. Cell morphology on SF was heterogeneous, comprising both spindle-shaped clusters with limited cell–cell contacts and occasional spherical aggregates. On BP, the morphology was more variable, with cells ranging from rounded to uniformly elongated, and the distribution of fluorescent markers was correspondingly irregular.

Collectively, these findings indicate that the SF patch possesses matrix properties and adhesive capacity comparable to those of the decellularized BP patch, while supporting better EC viability.

### 3.7. Intravital Assessment of Carotid Artery Patency and Vascular Patch Remodeling

#### 3.7.1. Ultrasound Examination

Ultrasound examination of the carotid arteries was performed at 2, 6, and 12 months post-implantation to evaluate blood flow velocity (BFV, cm/s) and the graft–artery complex diameter (GCD, mm) in both experimental groups. Across all time points, the observed values for both parameters were comparable between the SF and BP groups (Figure 6a, Table 3).

At each follow-up visit, the BP group exhibited slightly higher median BFV values compared to the SF group; however, the interquartile ranges overlapped considerably between the groups (Table 3). Regarding GCD, the median diameter in the SF group was somewhat greater than in the BP group at 2 months, but by 6 and 12 months, the intergroup differences had diminished to a minimal level (Table 3).

Analysis within the SF group revealed a gradual decline in both BFV and GCD from 2 to 12 months. In contrast, the BP group maintained relatively stable values for both parameters throughout the observation period (Table 3).

Despite this, statistical testing did not reveal any significant differences, either between the two groups at any given time point or within each group over time (Kruskal–Wallis test, *p* > 0.05 for all comparisons). Collectively, these findings indicate that both types of patches exhibited comparable hemodynamic and structural characteristics at all examined time points.

#### 3.7.2. Intravascular OCT Assessment of Neointimal Formation

OCT imaging confirmed neointimal formation on the luminal surface of both SF and BP patches at all examined time points (2, 6, and 12 months post-implantation). At the 2-month stage, the neointimal layer on the SF patch appeared more continuous and uniformly distributed across the graft surface, whereas on the BP patch it was heterogeneous and predominantly localized to the anastomotic zones (Figure 6b). Quantitative analysis at 2 months revealed minimal neointimal thickness in both groups; however, a marked intergroup difference was observed, with the BP group showing values nearly fivefold lower than those recorded for the SF group (Figure 6b, Table 3).

By 6 months post-implantation, the neointimal thickness in the SF group had increased more than 2.5-fold compared to the 2-month time point (*p* < 0.01). In contrast, the BP group exhibited a 30-fold increase relative to the previous time point (*p* < 0.01), resulting in neointimal thickness values that surpassed those of the SF group by the 6-month mark (Table 3).

At 12 months, the SF group showed a further 2.7-fold increase in neointimal thickness compared to the 6-month values (*p* < 0.01), while the BP group demonstrated only a modest, albeit statistically significant, increment (*p* < 0.01). By the end of the 12-month observation period, both materials exhibited comparable absolute neointimal thickness values (Table 3). Full quantitative measurements are provided in Table 3; representative OCT images are shown in Figure 6b.

### 3.8. Morphological Evaluation of Explanted SF Patches

#### 3.8.1. Two-Month SF Implantation Period

Morphological analysis of the explants confirmed the in vivo findings, revealing early integration of the SF patch into the surrounding tissues. The implantation site was surrounded by a connective tissue capsule without signs of inflammation. Macroscopically, the patch maintained its structural integrity without evidence of degradation or aneurysmal dilatation. Thanks to its pliable texture, the material acquired an appearance resembling that of a native artery. Transverse section analysis confirmed that the remodeled construct preserved a circular, patent lumen continuous with the adjacent vessel wall (Figure 7a).

Histological evaluation at 2 months revealed structural preservation of the SF patch, with no signs of biodegradation. A confluent, uniform neointima had formed, covered by a continuous monolayer of vWF-positive EC and containing α-SMA-positive cells within matrix rich in collagen III and IV. However, this neointimal layer lacked elastin fibers—a notable compositional divergence from the native arterial architecture.

The patch core was sparsely cellular, with cellularity increasing toward the anastomotic sites. The host response included the formation of a vascularized neoadventitial capsule rich in collagen III and IV. A limited foreign-body reaction, evidenced by giant cells and phagocytosis of fibroin, was confined to the patch periphery. Notably, no calcification was detected in the patch, supporting its biocompatibility and stability at this early time point, despite the observed elastin deficiency.

#### 3.8.2. Six-Month SF Implantation Period

At 6 months post-implantation, the SF patch remained intact, with focal areas showing ingrowth of host vascular tissue into the patch material (Figure 7a). Cellular infiltration within the patch core remained low, except at sites of host tissue ingrowth and along the neoadventitial border, where cellular accumulation was observed. The luminal surface of the artery overlying the SF patch was uniformly lined by CD31-positive and vWF-positive EC. The neointima was fully formed over the patch area and consisted of α-SMA-positive cells embedded in a collagen III and IV-rich matrix; its thickness had increased compared with the 2-month time point. The neoadventitia was composed of collagen fibers and fibroblast-like cells, with occasional vasa vasorum. No calcification foci were identified within the SF patch.

#### 3.8.3. Twelve-Month SF Implantation Period

Histological assessment at 12 months revealed advanced remodeling of the SF patch toward a trilamellar structure, comprising the neointima, residual patch material, and neoadventitia (Figure 7a). A markedly hyperplastic neointima, lined by vWF-positive EC and composed of α-SMA-positive cells, had developed; however, it notably lacked elastin, a key component of native arteries (Figure 7a). The patch matrix exhibited marked reorganization, including separation of fibroin layers, extensive cellular infiltration (macrophages, fibroblasts, smooth muscle cells), collagen deposition, and formation of vasa vasorum.

The surrounding neoadventitia increased in thickness compared with earlier time points and was populated by fibroblasts, collagen bundles, immune cells, and microvasculature. This progression suggests that host tissue integration and patch biodegradation resulted in a remodeled construct, whose principal morphological limitation was the absence of an elastic lamina (Figure 7a).

### 3.9. Morphological Evaluation of Explanted BP Patches

#### 3.9.1. Two-Month BP Implantation Period

At surgical re-exposure to the carotid artery, no gross structural abnormalities, aneurysmal dilations, or hematomas were observed at the BP patch implantation site. A vascularized connective tissue capsule enveloped the implantation site (Figure 7b). The explanted BP patch remained elastic and retained its structural integrity.

Cross-sectional analysis revealed that the implanted BP patch maintained a round lumen continuous with the adjacent vessel wall (Figure 7b). The neointima in the grafted region was thin and heterogeneous: thinner at the mid-portion of the vessel and thicker at the anastomotic zones. Histologically, the BP patch preserved its collagen architecture at 2 months, with isolated areas of delamination observed along its entire length.

The neointimal thickness did not differ significantly between the BP and SF patches. However, endothelial-like cell coverage of the inner surface was less complete, with regions devoid of a continuous EC monolayer. vWF was expressed uniformly along the graft. Within the neointima, α-SMA-positive cells were detected, together with diffuse collagen III and IV. Cellular infiltration of the BP patch was low, and elastin fibers were not observed. The neoadventitia contained fibroblast-like cells interspersed among collagen types III and IV fibers; isolated multinucleated giant cells indicative of a foreign-body response were also present (Figure 7b). Occasional vasa vasorum were noted at the neoadventitial interface with the BP patch and in the anastomotic regions. The junction between the BP patch and the native arterial tissue was surrounded by inflammatory cell accumulation.

#### 3.9.2. Six-Month BP Implantation Period

At 6 months post-implantation, the BP patch retained its overall structure, although delamination persisted, particularly with the neointima and neoadventitia interfaces (Figure 7b). Remodeling in the BP patch implantation site by this time point increased total vessel wall thickness, indicating marked NH overlying the BP patch (Table 3, Figure 7b).

Detailed histological examination revealed no signs of active inflammation or calcification within the explanted BP patch. The luminal surface was lined by a loose, discontinuous EC layer. The neointima tended to thicken both at the patch center and at the anastomotic sites. The BP patch itself maintained its fibrous structure. Cellularity remained low; however, isolated multinucleated giant cells indicative of a foreign-body reaction were present along the neoadventitial border.

Immunofluorescence analysis revealed flattened, α-SMA-positive cells within the neointima (Figure 7b). The EC lining was loose and discontinuous, with weak vWF expression. Collagen III fibers were present in both the neointima and the neoadventitia. Collagen IV was detected in individual, strongly stained cells distributed across all layers.

#### 3.9.3. Twelve-Month BP Implantation Period

After 12 months of implantation, the BP patch retained its lamellar collagen structure. In several cases, however, host vascular tissue grew between the patch layers. The luminal surface of the patch was lined by CD31-positive and vWF-positive EC throughout its length (Figure 7b). The neointima was homogeneous, composed of α-SMA-positive cells and collagen III and IV fibers. Neointimal thickening was observed throughout the entire patch area (Table 3, Figure 7b). A notable finding at 12 months was the presence of focal calcification deposits within the BP patch (Figure 7b).

In summary, remodeling of the BP patches implanted in the sheep carotid artery resulted in the formation of neointima- and neoadventitia-like tissue; however, the absence of elastin suggests that this resulting construct does not fully replicate the native arterial wall. Key features of the BP patches included the presence of delamination, indicative of material degradation, and focal calcification. The NH observed at 6 and 12 months highlights the differences between the BP patches and the native carotid artery wall.

## 4. Discussion

The present study provides a comparative evaluation of a biodegradable SF-based vascular patch and a clinically established BP patch by combining in vitro characterization with long-term in vivo implantation in a sheep carotid artery model. Our findings indicate that the SF patch exhibited uniaxial tensile properties comparable to those of native arteries, demonstrated acceptable hemocompatibility (lower hemolysis and lower platelet adhesion), showed cytocompatibility comparable to that of BP, and supported the formation of neointima- and neoadventitia-like tissue, although the absence of elastin suggests that the remodeled construct does not fully replicate a native artery. However, NH at 12 months in both groups highlights the need for further optimization.

The electrospinning and cross-linking methods employed in this study produced nanofibrous, microporous SF matrices with tensile properties—maximum tensile stress, force at failure, elongation at break, and Young’s modulus—comparable to those of native arteries under uniaxial tensile testing. Although these findings are encouraging, they represent only initial mechanical evaluation; other clinically relevant properties, such as burst pressure, suture retention strength, and cyclic fatigue resistance, were not assessed. In terms of hemocompatibility, SF adsorbed significantly greater amounts of albumin and fibrinogen than BP patches, but induced only minimal hemolysis and low platelet adhesion and activation. Maximum platelet aggregation, however, did not differ significantly between the two materials. These findings are consistent with the notion that protein adsorption alone does not predict thrombogenic potential; rather, the conformational state of adsorbed proteins and their subsequent cellular interactions determine the biological response [16]. Overall, SF exhibited favorable hemocompatibility in specific respects—particularly minimal hemolysis and reduced platelet adhesion—whereas its higher fibrinogen adsorption, despite comparable platelet aggregation, warrants further investigation.

The hydrolytic stability of SF was notable, with only 5.41% mass loss and a modest pH decrease to 6.56 over 12 months, despite its porous, biodegradable nature. This degradation profile closely parallels that of slowly degrading poly(ε-caprolactone) (2% mass loss over 21 weeks) and contrasts sharply with poly(L-lactide), which loses 30% of its mass within 13 weeks and acidifies the medium to pH 2.5–3 [17]. However, it should be acknowledged that the degradation test was performed under static conditions without medium replacement, which may cause local saturation with degradation products and a consequent pH decrease, as observed here; this should be considered when extrapolating the results to the dynamic in vivo environment. Nevertheless, the slow degradation of SF provides sufficient time for progressive material resorption synchronized with host tissue formation, which is a critical requirement for vascular grafts that must transition from prosthetic support to a neoartery. Importantly, in vivo implantation confirmed that SF does not undergo premature degradation; areas of bioresorption were progressively replaced by host tissue, indicating synchronization of degradation and neotissue formation.

Implantation into sheep carotid arteries demonstrated endothelial-like cell coverage and the formation of a trilaminar tissue organization consisting of neointima, residual patch material, and neoadventitia. Endothelial-like cell coverage of the luminal surface, confirmed by vWF and CD31 positivity, suggests the affinity of SF for host EC, corroborating the in vitro cytocompatibility data. However, functional assessment of the endothelial layer (e.g., barrier integrity, nitric oxide production, shear stress response) has not yet been performed, which remains a limitation. The formation of neointima and neoadventitia, together with vasa vasorum and extracellular matrix deposition (collagen types I, III, and IV), reflects the progressive integration of the SF patch with the host vascular wall. This integration pattern suggests that SF supports vascular tissue regeneration rather than merely serving as a permanent scaffold.

Although BP is the clinical standard for carotid patch angioplasty [18], it exhibited notable degenerative changes in this study. Consistent with previous reports [19,20,21], our morphological examination of explanted BP patches revealed collagen fragmentation, delamination, and isolated calcification foci at suture puncture sites at 12 months. These changes are attributed to fixation, which generates pyridinium rings that serve as nucleation sites for calcium phosphate deposition [20,21]. The presence of calcification and a marked connective tissue capsule around BP implants may predispose to sclerosis and impair long-term graft function.

A key finding was the development of NH, which was observed at 12 months in the SF group but was already evident at 6 months in the BP group. Intravital OCT confirmed arterial wall thickening in the implantation site, consistent with previous observations in both biodegradable and xenogeneic vascular grafts [22].

Several mechanisms may contribute to the NH observed in the present study, although these mechanisms were not directly investigated. The greater stiffness of SF and BP patches compared with that of native arteries may disrupt laminar blood flow, thereby causing endothelial injury and activating pro-oxidant, pro-inflammatory, pro-coagulant, and pro-apoptotic signaling pathways in adjacent smooth muscle cells (SMCs) [23,24]. In addition, endothelial dysfunction at the implantation site may impair endothelial nitric oxide (NO) synthase activity and reduce nitric oxide production [25,26]. A concomitant inflammatory response, characterized by leukocyte recruitment [27,28] and by activation of matrix metalloproteinases (MMPs) that degrade extracellular matrix components [29,30], may promote SMC migration and proliferation, ultimately leading to NH. In support of SMC involvement, immunofluorescence staining consistently revealed cells positive for α-SMA within the neointima at 2, 6, and 12 months. The earlier onset of NH in the BP group may be attributable to material degradation products or to the inflammatory response to xenogeneic collagen, both of which may accelerate vascular remodeling. Direct assessment of endothelial function, NO bioavailability, inflammatory mediators, and MMP activity was beyond the scope of this study; these parameters warrant future investigation.

The observed NH in both groups, particularly the later onset in the SF group, highlights a key challenge for biodegradable vascular grafts—the need to balance sufficient mechanical support with controlled degradation that permits tissue remodeling without triggering excessive intimal thickening. Although the SF patch supported endothelial-like cell coverage and the formation of neointima- and neoadventitia-like tissue, the absence of elastin and the development of NH at 12 months indicate that the remodeled construct does not fully replicate a native artery, and further material optimization is therefore required.

Potential strategies for reducing NH in SF-based grafts include surface modification with bioactive molecules (e.g., heparin, NO donors, or antiproliferative agents), tuning degradation kinetics to better match tissue regeneration, or incorporation of immunomodulatory cues to mitigate the inflammatory response. Future studies should explore these approaches and evaluate long-term outcomes in large animal models.

Several limitations should be acknowledged. The relatively small sample size (n = 6 per group) and the lack of analysis of signaling pathways involved in hyperplasia limit the generalizability of our findings. Additionally, the 12-month observation period, while substantial, may be insufficient to capture late-stage calcification or graft failure. Future studies with extended follow-up and mechanistic investigations are warranted.

### Limitations

The mechanical characterization was limited to uniaxial tensile testing without assessment of other clinically relevant properties (burst pressure, suture retention strength, cyclic fatigue resistance, compliance, biaxial behavior); therefore, the conclusion that SF is mechanically suitable for arterial reconstruction should be interpreted with caution and requires validation in future comprehensive testing.

The in vitro degradation assessment was performed in saline at 37 °C and pH 7.4 and therefore does not reproduce the enzymatic, inflammatory, cellular, protein-rich, or mechanical environment encountered in vivo; the results should be interpreted as an in vitro hydrolytic stability assessment rather than a direct predictor of in vivo degradation kinetics.

The limitations of this study are primarily due to the small sample size (n = 6 animals per time point, 18 animals in total; n = 2 for in vitro biocompatibility assays) without a formal power analysis, which may limit the generalizability of the findings. Although the paired implantation design (SF and BP patches in the contralateral carotid arteries of the same animal) reduced inter-animal variability, the longitudinal measurements at 2, 6, and 12 months were not analyzed using repeated-measures or mixed-effects models, and within-animal dependence across time points cannot be fully excluded. Additionally, only female sheep were used, and the 12-month follow-up may not capture late-stage complications, warranting future studies with larger cohorts, longer observation periods, and appropriate statistical modeling of repeated observations.

## 5. Conclusions

This study provides a comparative evaluation of a biodegradable SF-based vascular patch with a clinically established BP patch, by combining in vitro and in vivo approaches. The electrospinning and cross-linking methods employed produced nanofibrous, microporous SF matrices with an ultrastructure conducive to cell colonization and host tissue integration.

In vitro testing demonstrated that the SF patch exhibited tensile properties comparable to those of native arteries under uniaxial tensile testing, while its hemocompatibility was favorable in specific respects (lower hemolysis and lower platelet adhesion and activation compared with BP) and its cytocompatibility was comparable with that of BP.

Although implantation into the sheep carotid artery model resulted in endothelial-like cell coverage and the formation of neointima- and neoadventitia-like tissue, the absence of elastin and the development of NH at 12 months indicate that the remodeled construct does not fully replicate a native artery and that further material optimization is required to enhance the long-term performance of SF-based biodegradable vascular grafts.

## Figures and Tables

**Figure 1:**
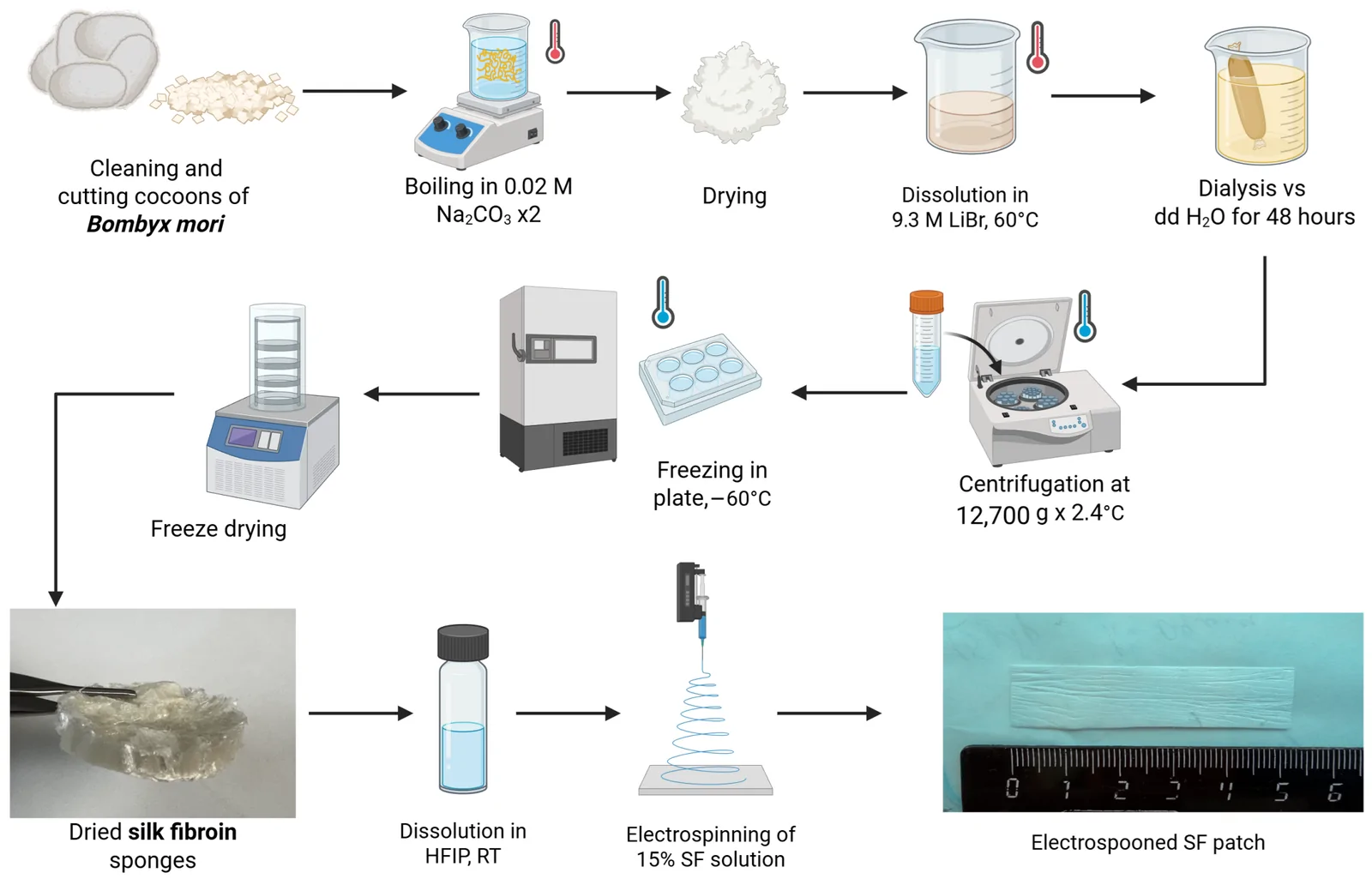
Scheme for the isolation of SF from cocoons of *Bombyx mori* and SF patch fabrication. Created in BioRender. Prokudina, E. (2026) https://BioRender.com/yzsawzn (accessed on 7 August 2026).

**Figure 2:**
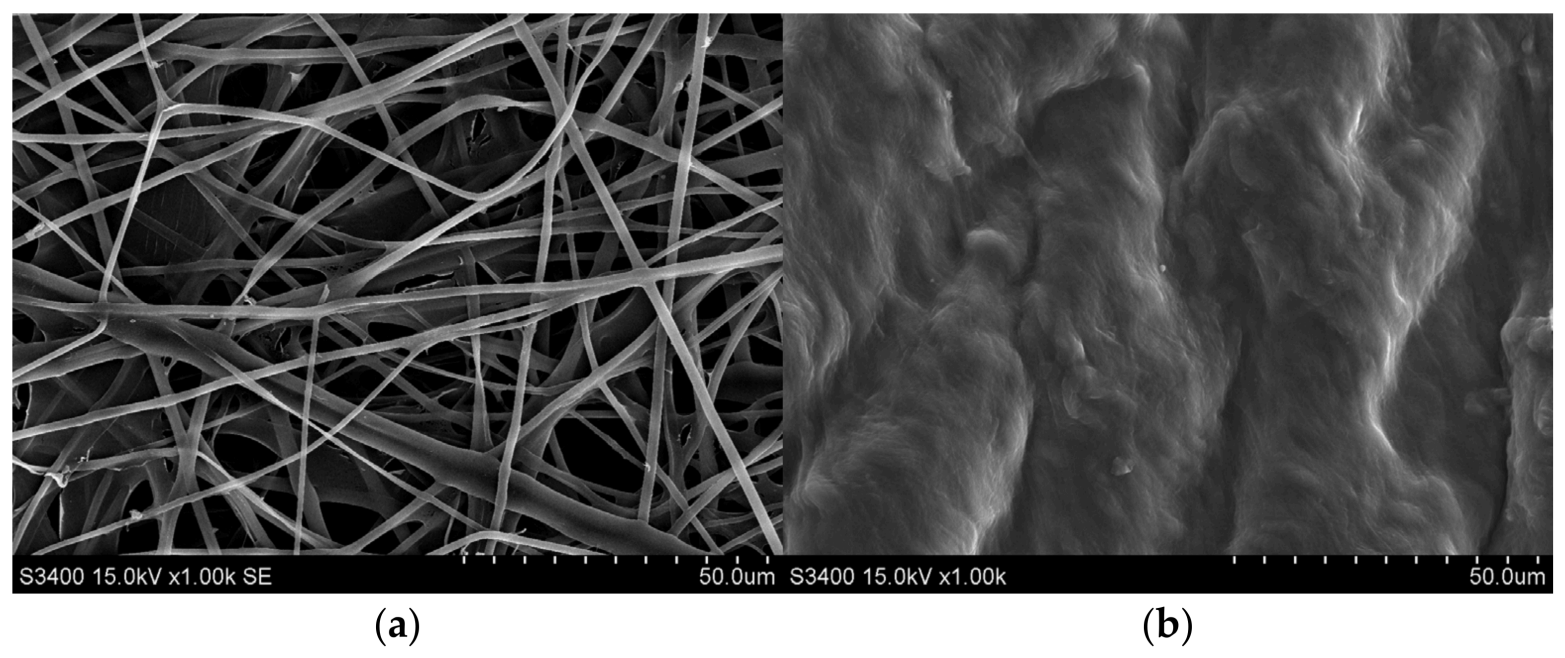
Surface ultrastructure of SF and BP patches: (**a**) scanning electron microscopy (SEM) image of the SF patch surface, showing the fibrous network structure (magnification ×1000); (**b**) SEM image of the serous surface of the BP patch, revealing its native collagen architecture (magnification ×1000).

**Figure 3:**
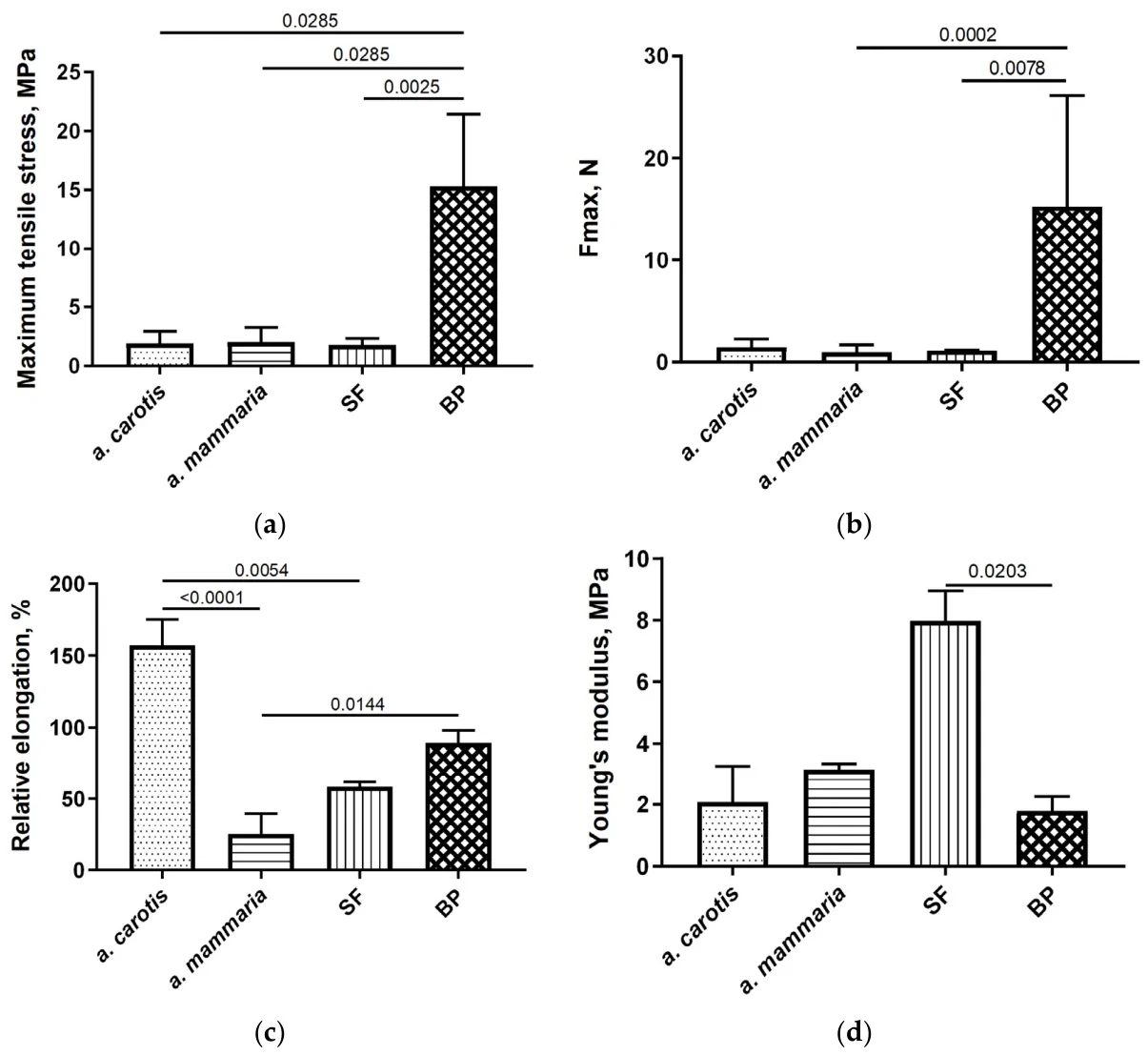
Uniaxial tensile properties of patches: (**a**) maximum tensile stress (MPa); (**b**) force at failure (Fmax, N); (**c**) relative elongation at break (%); (**d**) Young’s modulus (MPa). Data are presented as mean ± SD. SF, silk fibroin; BP, bovine pericardium.

**Figure 4:**
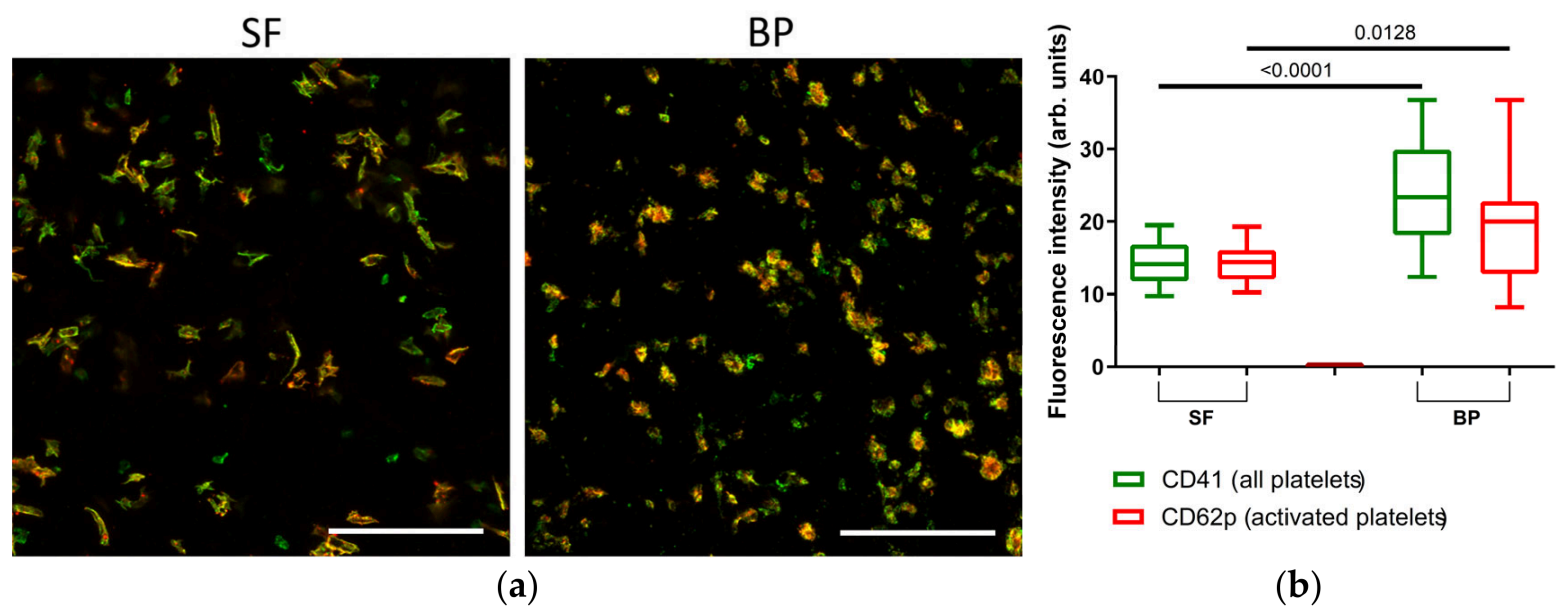
In vitro hemocompatibility of SF and BP patches: (**a**) confocal microscopy images of platelet adhesion and activation on the patch surfaces: green—CD41 (platelet marker), red—CD62p (activation marker), yellow—colocalization of both signals. Scale bar: 50 µm; (**b**) quantitative analysis of CD41 and CD62p fluorescence intensity (arbitrary units).

**Figure 5:**
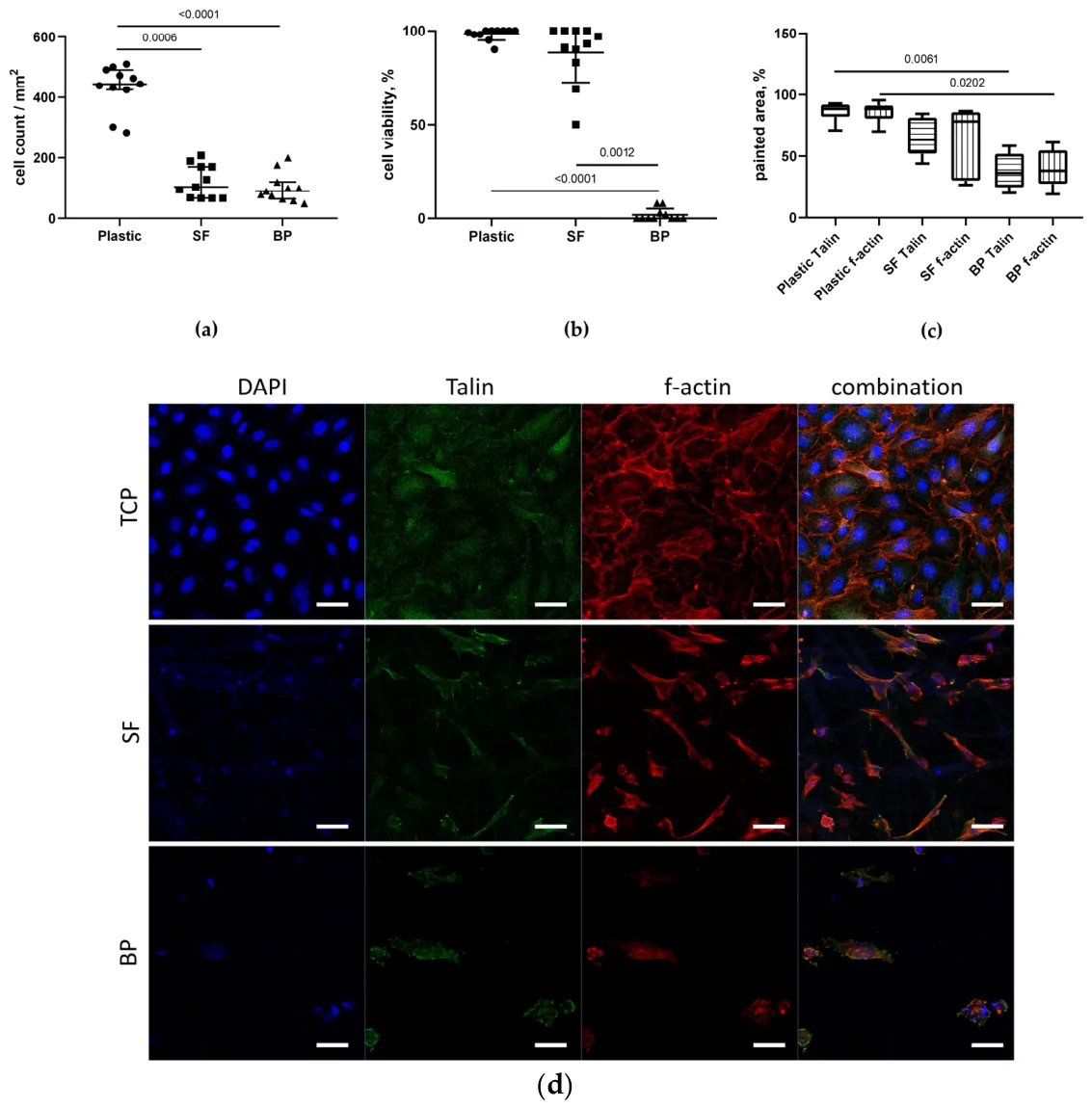
In vitro biocompatibility of SF and BP patches: (**a**) endothelial cell (Ea.hy 926) density (cells/mm^2^) on the patch surfaces and tissue culture plastic (control) after 72 h of culture; (**b**) Cell viability (%) on the same substrates; (**c**) relative area occupied by Talin-positive focal adhesions and F-actin cytoskeleton (% of total cell area); (**d**) confocal microscopy of Ea.hy 926 cells cultured on the patches: blue—DAPI (nuclei), green—Talin (focal adhesions), red—F-actin (cytoskeleton). Scale bar: 50 µm (Prokudina, E.S. et al. [15]). Data are presented as median with interquartile range (C–E); *p* < 0.05 vs. corresponding control.

**Figure 6:**
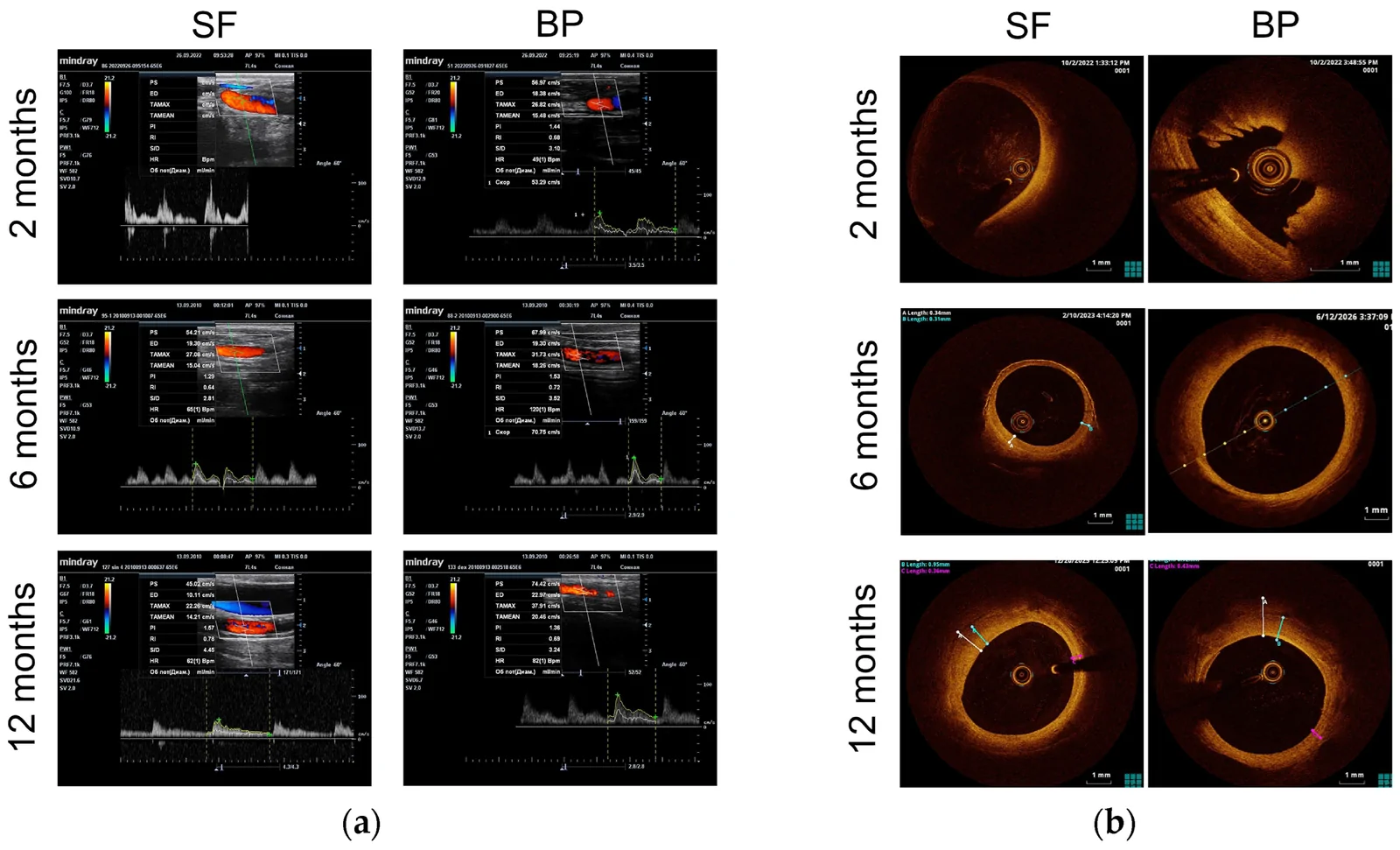
In vivo examination of carotid artery patency and patch remodeling: (**a**) duplex ultrasound imaging of carotid arteries at 2, 6, and 12 months post-implantation; (**b**) intravascular optical coherence tomography (OCT) of the implanted vascular patches. SF, silk fibroin; BP, bovine pericardium.

**Figure 7:**
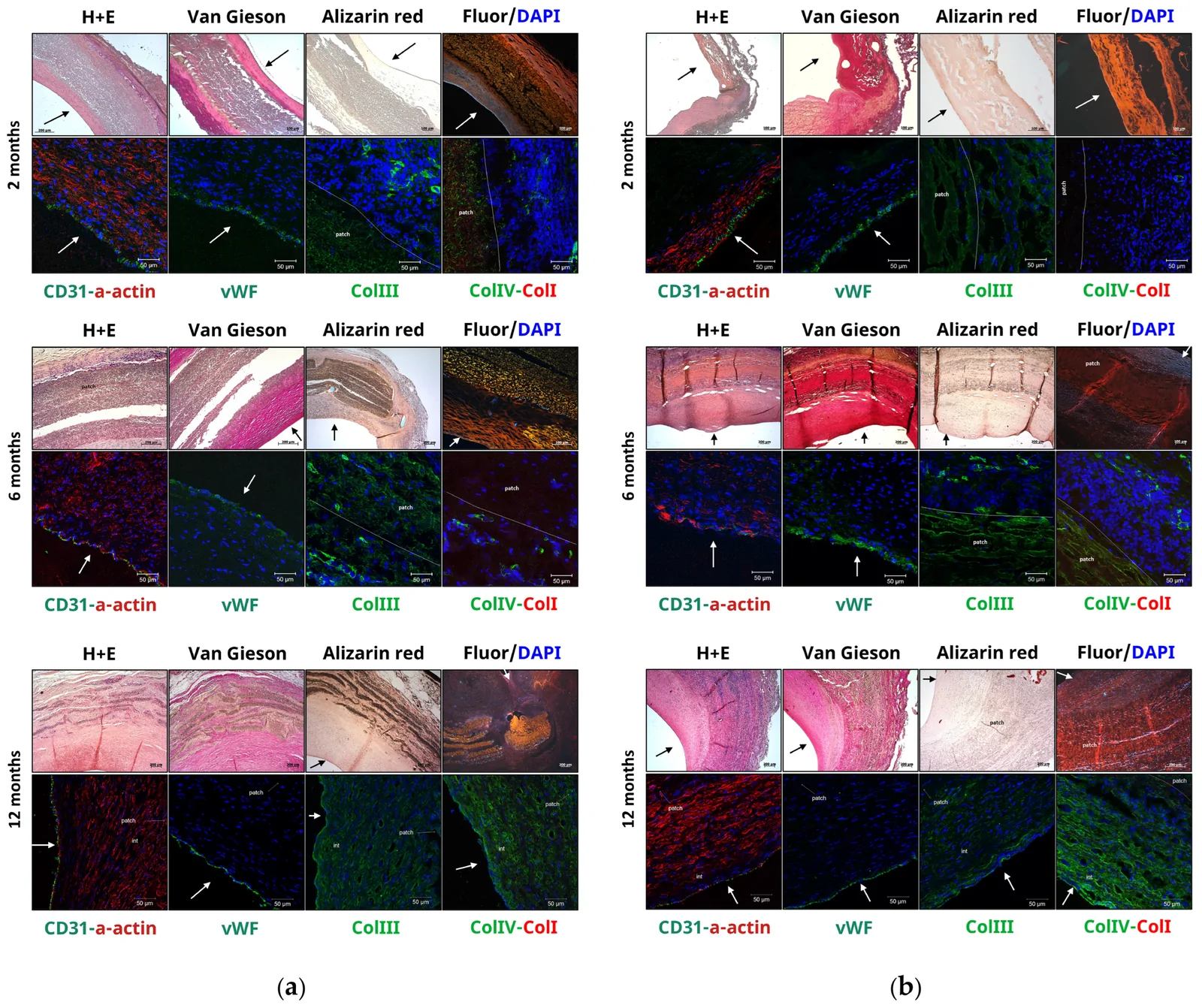
Histological and immunofluorescence analysis of explanted SF (**a**) and BP (**b**) patches at 2, 6, and 12 months after implantation into sheep carotid arteries. Light microscopy panels: hematoxylin and eosin (H&E) staining for general tissue morphology; van Gieson’s stain for collagen fibers; alizarin red C staining for calcium deposits. Confocal microscopy panels: immunofluorescence staining showing DAPI (blue, nuclei), CD31 (green, EC), α-SMA (red, smooth muscle cells), vWF (green, EC), collagen type III (green), collagen type IV (green), and collagen type I (red). Magnification: ×50, ×100, and ×200. Arrows indicate the luminal surface of the vessel. Scale bars are as indicated in the images.

**Table 1:** Protein elution from the patch materials after incubation with albumin or fibrinogen solutions.

Material	Albumin (mg/mL)	Fibrinogen (mg/mL)
SF	198.2 [176.8; 221.5]	469.3 [440.3; 498.7]
BP	100.0 [87.7; 112.4] *	77.3 [63.2; 91.3] *

*—*p* < 0.05 vs. SF (Mann–Whitney U test). Data are presented as median with interquartile range [Q1; Q3].

**Table 2:** Hemolysis and platelet aggregation parameters for SF and BP patches.

Material	Hemolysis (%)	Maximum Platelet Aggregation (%)
SF BP	0.006 [0.004; 0.024] 0.020 [0.016; 0.025] *	86.4 [84.5; 90.0] 85.0 [83.6; 87.2]
PRP	-	84.0 [81.8; 86.1]

*—*p* < 0.05 vs. SF (Mann–Whitney U test). Data are presented as median with interquartile range [Q1; Q3].

**Table 3:** Blood flow velocity (BFV), graft-artery complex diameter (GCD) and neointimal thickness in SF and BP groups at 2, 6, and 12 months after implantation.

Parameter/Group	2 Months	6 Months	12 Months
BFV (cm/s) SF BP			
	58.0 [43.5; 74.0]	55.5 [50.0; 61.0]	51.0 [45.0; 57.0]
	69.0 [60.1; 81.7]	67.5 [60.8; 80.5]	62.8 [54.4; 72.3]
GCD (mm) SF BP			
	5.4 [4.4; 5.8]	4.7 [4.1; 5.7]	4.4 [4.0; 5.1]
	4.9 [4.4; 5.1]	5.0 [4.5; 5.6]	4.9 [4.5; 6.1]
Neointimal thickness (µm) SF BP			
	130.7 [123.8; 139.1] *	332.5 [251.2; 376.3]	886.6 [820.4; 948.8] *
	26.9 [23.3; 36.8] **	817.7 [730.8; 828.2] *	890.7 [858.3; 939.4] **

*—*p* < 0.01 vs. SF 6 months; **—*p* < 0.01 vs. BP 6 months. Data are presented as median with interquartile range [Q1; Q3].

## Data Availability

The original contributions presented in this study are included in the article. Further inquiries can be directed to the corresponding author.

## Abbreviations

The following abbreviations are used in this manuscript:

SF	Silk fibroin
BP	Bovine pericardium
OCT	Optical coherence tomography
NH	Neointimal hyperplasia
ePTFE	Expanded polytetrafluoroethylene
EC	Endothelial cells
SEM	Scanning electron microscopy
BSA	Bovine serum albumin
PBS	Phosphate-buffered saline
SDS	Sodium dodecyl sulfate
ADP	Adenosine diphosphate
PRP	Platelet-rich plasma
PPP	Platelet-poor plasma
TCP	Tissue culture plastic
DAPI	4′,6-diamidino-2-phenylindole
H&E	Hematoxylin and eosin
α-SMA	α-smooth muscle actin
vWF	Von Willebrand factor
BFV	Blood flow velocity
GCD	Graft–artery complex diameter
SMCs	Smooth muscle cells
